# Racial discrimination in surgery: A systematic review

**DOI:** 10.1007/s13304-023-01491-x

**Published:** 2023-03-10

**Authors:** Michael El Boghdady, Beatrice Marianne Ewalds-Kvist

**Affiliations:** 1 Department of General Surgery, Guy’s and St Thomas’ NHS Trust, London, UK; 2 Equality and Diversity Officer, Association of Surgeons in Training, London, UK; 3 University of Edinburgh, Edinburgh, United Kingdom; 4 Stockholm University, Stockholm, Sweden; 5 University of Turku, Turku, Finland

**Keywords:** Surgery, Racial discrimination, Racism, Burnout

## Abstract

**Introduction:**

Racial/ethnic discrimination indicates the stereotyped or unkind conduct of superiority towards other persons based on their race or skin color. The UK General Medical Council published a statement supporting zero-tolerance approach to racism in the workplace. We aimed to systematically review racial discrimination in surgery and answer the following questions: (1) Does racial/ethnic discrimination in surgery exist in citations from the last 5 years. (2) If yes, are ways suggested to reduce racial/ethnic discrimination in surgery?

**Methods:**

The systematic review was performed in compliance with the PRISMA guidelines along AMSTAR 2. A 5-year literature search was carried out on PubMed for articles published from 1/1/2017 to 01/11/2022. Search terms were ‘racial discrimination and surgery’, ‘racism OR discrimination AND surgery’, ‘racism OR discrimination AND surgical education’. The retrieved citations were quality assessed by MERSQI and evidence graded by GRADE.

**Results:**

A total of 9116 participants responded with a mean of 1013 (SD = 2408) responses per citations reported in 9 studies from a final list of 10 included citations. Nine studies were from USA and 1 from South Africa. There was evidence of racial discrimination in the last 5 years and the results were justified on strong scientific evidence constituting the basis for evidence grade I. The second question’s answer was ‘yes’ which was defendable on moderate scientific recommendation and thereby establishing the basis for evidence grade II.

**Conclusion:**

There was sufficient evidence for the presence of racial discrimination in surgical practice in the last 5 years. Ways to decrease racial discrimination in surgery exist. Healthcare and training systems must increase the awareness of these issues to eliminate the harmful effect on the individual as well as on the level of the surgical team performance. The existence of the discussed problems must be managed in more countries with diverse healthcare systems.

## Introduction

The term “racism” refers to prejudice defining that one person feels superior over another. Racial discrimination indicates the stereotyped or unkind conduct towards other persons based on their race or skin color [1]. It was reported that black doctors were six times less likely to get a job in London, and Asian doctors were four times less likely than white applicants [2]. Ethnic-minority doctors are also less likely to pass postgraduate examinations – termed “differential attainment” which was defined as the gap in achievement between different demographic groups undertaking the same assessment [3]. Differential attainment by ethnicity has existed in the medical workforce throughout each measure of training and career progression [4]. Doctors from ethnic minorities were reported to be more likely to face referral to the General Medical Council in the UK, more likely to have their cases investigated, and may face harsher sanctions after an investigation [5].

A previous study scrutinized in details stress neurobiology and mental aftermath of racial discrimination [6]. Racial/ethnic bias can create dysregulation in mediators of allostasis, such as altered hypothalamic–pituitary–adrenal-axis activity, altered heart rate variability, altered sympathetic nervous system and parasympathetic nervous system [6]. Another study examined the relationship between racial discrimination and real-time physiological stress responses and concluded that the parallel use of salivary biomarkers and ecological momentary assessments functioned to examine this temporal relationship [7]. Also, racial discrimination’s harmful health effects in ethnic minority have been validated [8]. Racial bias does harm both health and simultaneously the length of the lifetime because racial bias due to the repeated and chronic stress accumulates from allostatic load to allostatic overload with extra costs for the body from increased endocrine or neural response intensity [9].

Based on both psychological and physical harmful stress responses towards unethical conduct, we aimed to study if racial discrimination still exists in surgical practice. Therefore, our research questions were as follows:

A. Does racial/ethnic discrimination in surgery exist in citations from the last 5 years?
B. Are ways suggested to reduce racial/ethnic discrimination in surgery?

## Methods

A systematic review was performed in compliance with the PRISMA (preferred reporting items for systematic review and meta-analysis) guidelines [10] along AMSTAR 2 [11] and GRADE recommendations [12].

### Search strategy

**Fig. 1:**
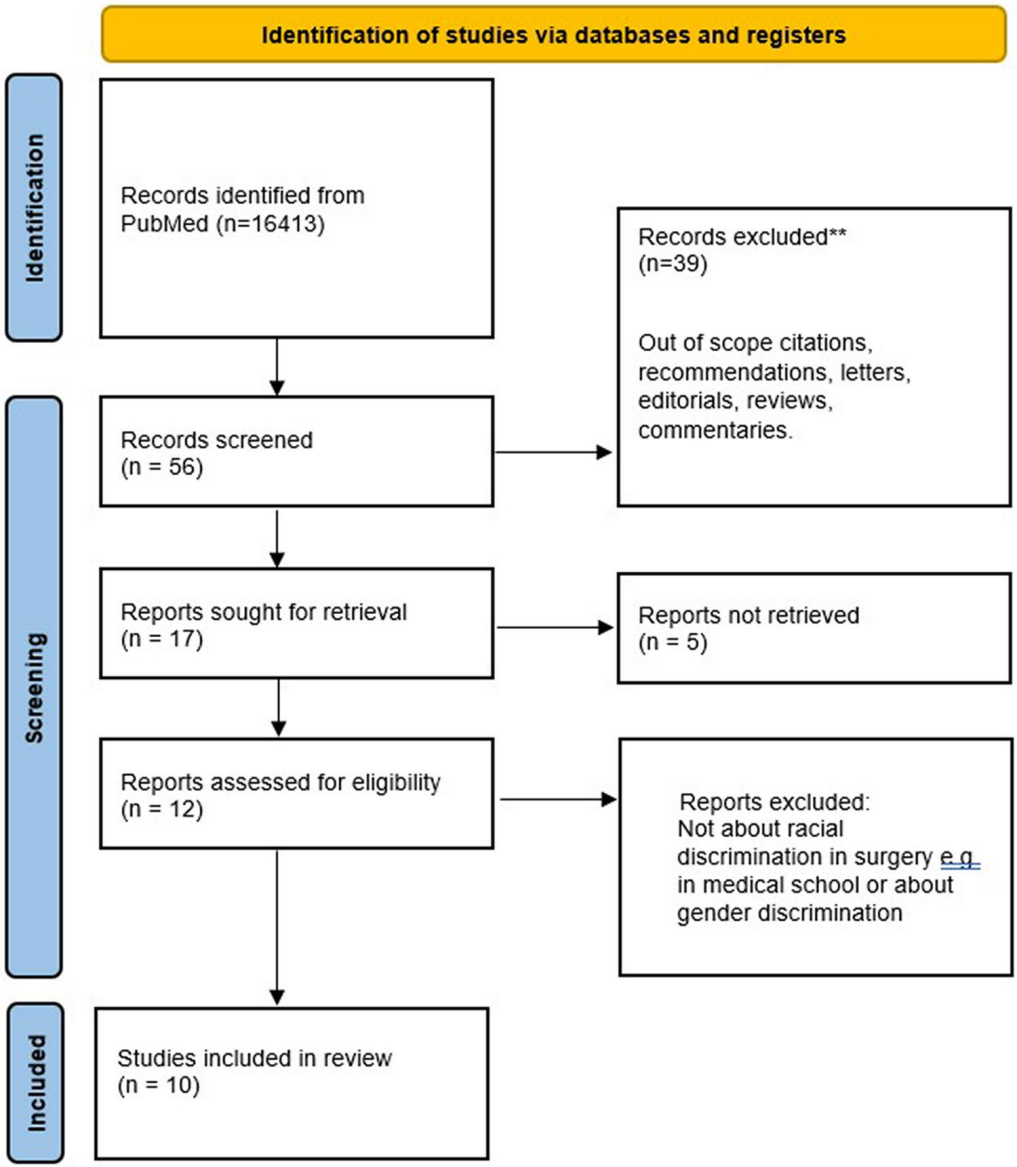
PRISMA flow diagram of the included citations

A 5-year literature search was carried out on PubMed for articles published from 1/1/2017 to 01/11/2022 (Fig. 1). Search terms were: ‘’racial discrimination and surgery’’, ‘’racism OR discrimination AND surgery’’, ‘’racism OR discrimination AND surgical education’’.

### Inclusion and exclusion criteria

Only quantitative publications related to racial/ethnic discrimination and surgery were included in this study. Conference abstracts, letters, editorials, commentaries, protocols, reviews, qualitative papers, and non-English publications were excluded.

### Procedure

The procedure for developing a systematic review comprised two authors’ inspection of titles, abstracts, and full-text papers, which were systematically reviewed against the inclusion and exclusion criteria. The detailed literature search was independently performed. The final list of citations was completed by both authors. Search items were studied from the nature of the article, date of publication, and aim as well as main findings in relation to racial discrimination and surgery.

### Contribution to synthesis

Studies were grouped for the synthesis after quality-assessment outcome according to their scientific-value contribution to a certain evidence grade.

### Quality assessment

The retrieved citations were available in full text. Quality assessment of the citations was applied using The Medical Education Research Study Quality Instrument (MERSQI) which contains 10 items that reflect 6 domains of study quality including study design, sampling, type of data, validity, level of data analysis, and outcomes [13]. Each dimension could contribute to the final score with a maximum of 3 scores in case they apply to the citation’s content. For the assessment of the validity of evaluation instrument, we focused on face validity, limitations or reliability, and correlations with other methods. MERSQI score represents the adjusted mean of two assessors’ quality estimations of each citation and the interrater reliability of the two assessors was significant (*r*[10] = 0.78; *p* < 0.01, two tailed and r_s_ [10] = 0.71; *p* = 0.02, two tailed, respectively). MERSQI generates a potential range from 5 to 18 scores depending on the dimensions’ correspondence with the content of the citation. At present, a range from 9 to 13.5 scores was produced depending on the dimensions’ degree of correspondence to each of the 10 citations.

Our method for identifying and evaluating data has been reported in line with assessing the methodological quality of systematic reviews (AMSTAR 2). There was a good compliance with Amstar 2 tool. Reporting ‘’yes’’ in 11 criteria and ‘’partial yes’’ in one. The 4 ‘’no’’ were related to meta-analysis, which was not applicable in this study.

### Evidence grading

Quality of evidence for the evidence grading was based on criteria for using GRADE [12], comprising four grades:

Evidence grade I: strong scientific evidence based on at least 2 studies with high evidential value or a systematic review/meta-analysis with high evidential value.

Evidence grade II: moderate scientific basis: a study with high evidential value and at least 2 studies with moderate evidential value

Evidence grade III: low scientific evidence: a study with high evidential value or at least 2 studies with moderate evidence value

Evidence grade IV: insufficient scientific evidence: 1 study with moderate evidence and/or 2 at least 2 studies with low evidential value

### Risk of bias within and across studies

We assessed the risk of bias in a blind manner and the quality estimates were completed by the two authors, separately. If the assessments did not agree, we adjusted and averaged the scores of the two quality assessors. The means were by visual binning divided into 4 classes, but we discarded classes of low or insufficient scientific evidence in our result. Thereby, we regarded that we filled the GRADE’s criteria for consideration of an individual study’s risk of bias or quality to estimate the study’s suitability for establishing a basis for the ratings about its strength to contribute to the body of evidence of the findings in this systematic review. Thereafter, we controlled for accumulated risk of bias by calculating and grading the body of evidence after the quality-assessment outcome according to PRISMA’s recommendations.

### Statistics

Descriptive statistics like Pearsons r and Spearmans rho were computed by IBM SPSS version 26.

## Results

After a careful selection procedure, a final 10 citations were included in our systematic review. A total of 9116 participants responded with a mean of 1013 (SD = 2408) responses per citations reported in 9 citations. Nine studies came from USA and 1 from South Africa. Altogether, 8 cross-sectional studies, 1 prospective cross-over trial cluster-randomized study as well as 1 retrospective cross-sectional analysis were included in the results [14–23]. The latter citation acknowledged only percentages as opposed to number of participants. Furthermore, the 10 citations’ mean quality score was computed to be 11.33 (SD = 1.73). The limits of the four quality classes were established by visual binning as follows: <  = 9,63 scores denoted insufficient quality (IS) of a citation. A range from 9.64 to 10,25 scores represented low quality (L) and scores from 10,26 to 12,50 signified moderate quality (M) and scores ranging from 12,51 to 13,50 denoted high quality (H). Altogether, there were 2 IS, 3 L, 2 M and 3 H. We discarded citations of low and insufficient quality (Table 1).

**Table 1:** Tabular analysis of included citations

Author & Jounral	Type of study	Aim of study	Participants, specialty & Country	Outcome	Q-s^1^ *X* =	E-gr^2^
Blum JD et al. [14] J Cranio-fac Surg	Cross- sectional study	To study diversity and provide an updated profile of the international cranio-maxillofacial workforce along with perceived discrimination	91 cranio-maxillo-facial surgeons (CMFS) 2/3 of participants practised in US Philadelphia, USA	Most respondents were White (n = 73), non-Hispanic (n = 85), hetero- sexual (n = 72), cisgender males (n = 74). Practice setting was mainly academic (n = 60) and group / hospital-based (n = 68). US CMFS reported more racial and sexual orientation-based bias than non-US surgeons	9,75 L	
Criddle TR et al. [15] J Oral Maxillo-fac Surg	Cross- sectional study	To explore factors why African Americans choose a career as an oral and maxillofacial surgeon (OMS); to examine satisfaction among minority OMS with the residency and training; to report on practice patterns among minority OMS and to identify bias for or against minority OMS to further the foster diversity	41 oral and maxillofacial surgeons (OMS) from Chicago, USA	Most respondents reported that race did not affect the application to a residency program as well as did not affect their practice. Yet, 25–46% of OMS had experienced race-related bias, and 48–55% met unfairness against African Americans in OMS	9 IS	
Fitzgerald CA et al. [16] Am Surg	Multicenter cross-sectional study	To evaluate the prevalence of bias and abuse among surgical residents by means of HITS (Hurt, Insulted, Threatened with harm or Screamed at) survey. Estimating the prevalence of bias, verbal abuse, and unfairness among residents is difficult as the episodes are underreported	76 surgical residents from California, Florida, Georgia, Ohio, Texas, Virginia USA	The HITS was positive in 3.9%. The abuse comprised sexual harassment (28.9%), gender bias (15.7%), and discrimination based on ethnicity (7.9%). Gender and racial bias correlated. Individuals who were insulted, were more likely to be physically threatened or verbally abused. Surgical residents in academic teaching hospitals across the United States are often subject to bias	10 L	
Hu YY et al. [17] N Engl J Med	Cross- sectional national study	To ask general surgery residents to report mistreatment often leading to burnout and suicidal thoughts during the past year. Mistreatment and burnout were assessed by modified Maslach Burnout Inventory	7409 (99.3%) general surgery residents from Chicago, Philadelphia USA	16.6% reported racial bias, 30.3% verbal or physical abuse, 31.9% gender bias, and 10.3% sexual harassment. 65.1% of the women reported gender bias and 19.9% sexual harassment. Patients and patients' families were regular sources of gender and racial bias (47.4%), whereas attending (consultant) surgeons were frequent sources of sexual harassment (27.2%) and abuse (51.9%). Weekly burnout symptoms had 38.5% of residents, and 4.5% had suicidal thoughts. Exposure to bias, abuse, or harassment a few times per month provoked signs of burnout and suicidal thoughts	13.5 H	E-GR ^=^ I
Khubchandani JA et al. [18] J Surg Res	Prospective cross-over trial cluster-randomized study	To study the extent of perceived discrimination among general surgery residents of color by completing the Everyday Discrimination Scale (EDS)	Out of 266 residents across seven residency programs in Boston, Massachusetts USA	Racial breakdown revealed 59% White, 17% Asian, 11% Black, and 5% Multiracial. A total of 22% fell into the High EDS score group. Resident race, fluency in a language other than English, and median household income were associated with EDS scores. Black residents were 4.2 times as likely to have High EDS scores than their White counterparts. Black surgical residents experience high levels of discrimination on a daily basis	12.75 M	E-GR ^=^ II
Naidu P et al. [19] S Afr J Surg	Cross-sectional national study	To uncover the barriers and trials that South African surgeons confront in their training and career	129 participants: Specialist surgeon 87 (71%) Registrar 23 (19%) Fellow 8 (7%) Medical officer in surgery 5 (4%) from Cape Town & KwaZulu-Natal, South Africa	111 surgeons stated that they did not repent practicing surgery but barriers comprised limited personal time, heavy surgical workload, and trouble taking vacation, restricted postgraduate training, and verbal discouragement. Challenges comprised work-life imbalance, racial biased and gender inequity and burnout	9,5 IS	
Pillado EB et al. [20] J Vasc Surg	Cross-sectional study	To characterize the kinds and sources of racial/ethnic discrimination among vascular surgery trainees. The primary outcome measures were self-reported mistreatment and causes of mistreatment between race and ethnicity groups	510 trainees from vascular surgery from Chicago, IL; New York and Syracuse, NY; San Francisco, CA; New Orleans, LA; and Ann Arbor, MI, USA	Black and Asian trainees were mistaken for a non-physician, subject to slurs or hurtful comments, isolation, or mistaken for a trainee of same race/ethnicity. Only 62.5% of Black trainees’ institution take the mistreatment seriously compared to White (88.9%), Hispanic/Latinx (88.2%), Asian (83.2%), and other/prefer not to say (71.4%) trainees	13 H	E-GR ^=^ I
Smith RM et al. [21] J Surg Educ	Experimental analysis of plastic-surgery education tools	To improve introductions to accurate representations of non-white skin tones for residents of Plastic surgery education. Evaluation done by using skin tone as proxy, race representation in images used in the American Society of Plastic Surgery Resident Education Curriculum	An average of 1861 color photographs and 237 graphics were rated by 6 reviewers by means of Fitzpatrick’s scale with skin tones Plastic surgery Minnesota, USA	A significant difference between images and graphics categorized as Fitzpatrick I to II and Fitzpatrick V to VI was found. While 76% of patients in US are white and 13% Black, the researchers demonstrated both an unequal and unrepresentative distribution of photos and graphics of non-white patients so plastic surgeons need to learn about all races’ skin tones	12,75 M	E-GR ^=^ II
Sudol NT et al. [22] JAMA Surg	Cross-sectional survey	To investigate microaggressions (MA) against racial/ethnic–minority surgeons and anesthesiologists along with sexist MA against the women. Further, it was described how MA links to severe distress leading to physician burnout. The Maslach Burnout Inventory, the Racial Microaggression Scale, and the Sexist Microaggression Experience and Stress Scale were used	652 surgeons and anesthesiologists Southern California Los Angeles USA	Racial/ethnic-minority surgeons and anesthesiologists were subject most commonly MA from a few leaders or co-workers of the same race/ethnicity. Underrepresented minorities (URM) were more likely to experience environmental inequities and criminality. Racial/ethnic–minority female physicians who experienced racial MA were more likely to report burnout; if they were subject to both racial/ethnic MA and sexism were even more likely to experience burnout. The overall prevalence of physician burnout was found to be 47%	13,25 H	E-GR ^=^ I
Zhu K et al. [23] J Surg Res	Retrospective cross-sectional analysis	To analyze and quantify the relationship of race and gender and academic rank, tenure status, and degree in American academic surgical faculty	White 69.8% Asian 15.8% Black 3.2% Hispanic 3.4% Multiple races 3.5% surgeons academic rank, tenure status, and degree in American academic surgical faculty British Columbia, Canada Kerala, India California, USA	There were more whites and men in surgery than other races and women. Asians and women increased across all ranks with 7% and 5% in full professor, 5% and 6% in associate professor. Women and Asians are increasing in relative representation; but racial and gender disparities remain prevalent at higher academic ranks and positions of leadership, especially among Black and Hispanic academic surgeons	10.75 L	

^1^Q-S^=^ Quality score

*X* = Mean

^2^E-GR = Evidence grade

Ten citations are described. Out of three high-quality citations, 2 support findings on evidence grade I and 1 + 2 moderate-quality papers support findings on evidence grade II

### Does racial/ethnic discrimination in surgery exists in citations from the last 5 years?

Hu et al. [17] indicated that around 50% of general surgery residents were subject to at least one form of mistreatment, with 19.0% mistreated a few times per month and 30.9% a few times per year. Racial discrimination was reported by 18.6% of women and 15.1% of men. Altogether 4.5% of residents had suicidal thoughts during the past 12 months. Bias due to pregnancy or childcare was reported by 7.2% of all residents. Racial discrimination was reported by 47.4% of residents to stem from patients and patients’ families as well as from attending surgeons (also known as consultants in UK) (17.4%), nurses and staff (10.7%), and other residents (8.2%). Discrimination due to pregnancy and childcare came from other surgeons: attendings (36.8%) and other residents (22.6%). Verbal or emotional abuse was reported by 30.2% of all residents and stemmed from other surgeons: attendings (52.4%) and other residents (20.2%). Physical abuse was sporadic (2.2%) and was reported equally by men and women. Each separate mistreatment measure was related to burnout. Symptoms of burnout were assessed by means of Maslach Burnout Inventory [MBI], [24, 25]. MBI comprises 3 subscales that signals that a person suffers from burnout as follows: Emotional exhaustion (EE) goes with feelings of like lack of control over what happens in life. Depersonalization (DP) goes with feelings that you are disconnected, you are an outside observer of your thoughts, feelings and body. Personal accomplishment (PA) goes with feelings of reduced competence, low self-efficacy, and reduced sense of achievement [26]. Burnout symptoms came to pass at least once a week and were reported by 38.5% of residents, with 34.3% reporting symptoms of emotional exhaustion (EE) at least weekly and 17.1% reporting symptoms of depersonalization (DP) at least weekly. In other words, general surgery residents were subject to much racial and other kinds of mistreatment.

Sudol et al. [22] revealed that racial/ethnic microaggressions were confronted by 299 of 367 racial/ethnic-minority physicians (81%). The most usual racial/ethnic-minority group was Asian, (females 39%). A total of 58 out of 588 individuals (10%) belonged to an underrepresented minority (URM) group with Black, Hispanic, or Hawaiian/Pacific Islander individuals. Female physicians were most targeted for microaggressions, particularly environmental or few role models, authority figures, and co-workers of the same race. Attitudes targeting “not a ‘true’ American” or being a “foreigner,” occurred in Asian, South Asian, and Middle Eastern physicians. By Maslach Burnout Inventory (MBI) physician burnout was assessed with 3 subscales: emotional exhaustion (EE), depersonalization (DP) and personal accomplishment (PA) [24, 25]. When stratified by race, PA was higher for White physicians (84%) compared with racial/ethnic-minority physicians (72%). Racial/ethnic-minority physicians were more likely to experience low PA compared with White physicians. Racial/ethnic-minority females who experienced racial/ethnic microaggressions were likely to report physician burnout. Furthermore, racial/ethnic-minority female physicians who experienced both racial/ethnic and sexist microaggressions were most likely to experience burnout compared with racial/ethnic-minority and White male physicians. The prevalence of racial/ethnic microaggressions against racial/ethnic-minority surgeons and anesthesiologists, with special bias against females, was high and associated with physician burnout. The authors provided evidence-based data on surgical workplace mistreatment [22].

To answer the question if racial/ethnic discrimination in surgery existed and still exists in the last 5 years, the answer is **yes.** The answer is justified on **Evidence** grade I, that is, strong scientific evidence based on 2 studies with high evidential value. In other words, Hu et al. [17] and Sudol et al. [22] constituted the basis for this conclusion (Table 1).

### Are ways suggested to reduce racial/ethnic discrimination in surgery?

Pillado et al. [20] studied 510 (85.9%) out of 594 U.S. vascular surgery trainees who completed the survey. They suggested that to reduce racial/ethnic discrimination in surgery, wrongdoers’ rehabilitation must happen. Wrongdoers must reconciliate with the sufferer and the whole community, which process is called ‘restorative justice’. This restorative justice [27] brings about an opening for those who have been discriminated as well as for those who have victimized others, thereby they can re-claim equity through unrestricted discussions about discrimination and accountability in treating other persons. Moreover, both parts can shift their focus to the needs of each person after the incident. The whole community can contribute to improvement for racial/ethnic minorities by economically supporting education about diversity, equity, and inclusion.

Khubchandani et al. [18] evaluated the Provider Awareness, and Cultural dexterity Toolkit for Surgeons (PACTS) curriculum and aimed at quantifying perceived discrimination among surgical residents by means of the Everyday Discrimination Scale (EDS) which ranged from 0 to 36 scores; higher scores represented more perceived bias (Md= 7 scores). The EDS scores were collapsed into a Low group (0-12), and a High group (13-36); 208 (78%) out of 266 residents reported Low EDS scores and 58 (22%) reported High scores. The researchers claimed that their study captures the level of discrimination among surgical residents both in and out of the hospital. Their remedy to deal with racism is to educate for example, the staff to fully understand the life experiences of surgical residents, to care about providers holistically, and to scrutinize the distress of provider discrimination on surgical care delivery, as well as to supervise resident mental-health wellbeing to avoid burnout and/or attrition. Institutional leaders’ increased awareness of these findings is a must as they strive to cultivate a diverse surgical training environment. It will also be important to understand the impact of perceived discrimination on personal healthiness, resident attitudes and knowledge about curing a diverse range of patients, and patient clinical consequences. Explicitly, it will be fundamental to understand how aftermaths of perceived discrimination influence adoption, and impact the PACTS curriculum [18].

Smith et al. [21] stated that racial-health implicit bias controls the healthcare system. Implicit bias means: “….embedded stereotypes affect decision-making without conscious thought” and is ever-present in medicine. The unconscious inclusion of white patients at the exclusion of Black patients, elevates one race over another and is named the “hidden curriculum” of medical education. White patients have come to represent the “normal” patient. Ultimately, this “Whiteness” fixation limits and can interfere with a physician’s ability to identify diseases in patients of color. The described underrepresentation among images and photographs of skin colors adds to implicit bias and to racial disparities within healthcare [28].

Even though considerable effort has reached the training physicians to educate them to be conscious of their own bias and also to be conscious about “embedded stereotypes” that exist and persist in the medical education in order to make meaningful changes, there are still work to do. The authors finish with the words: “Identifying and then confronting existing representation trends in images used in plastic-surgery education is one controllable, yet powerful technique that can make plastic surgeons leaders in the pursuit of an anti-racist medical culture.”[21].

The question: Are ways suggested to reduce racial/ethnic discrimination in surgery.can be answered with yes on a moderate scientific basis according to conditions for Evidence grade II requiring one study [29] with high evidential value and 2 studies with moderate evidential value [18, 21].

## Discussion

Racial/ethnic discrimination in surgery exists in citations from the last 5 years. During these 5 years more than 50% of all general surgery residents reported some form of mistreatment, the highest prevalence and severity existed among racial/ethnic-minority female physicians, explicitly among underrepresented minority (URM) physicians. Racial/ethnic-minority females with racial/ethnic microaggressions were likely to report physician burnout [17, 22]. Burnout is serious and assists as a precursor to suicidal ideation. Both research groups used MBI for assessment of physician burnout. MBI includes the subscales Emotional Exhaustion (EE), depersonalization and personal accomplishment. EE needs adequate coping strategies to deal with the problematic incidents. By use of productive strategies such as direct action and help seeking, lower levels of occur than by those who use escaping problems [29]. As a problem-focused strategy helps exercising self-awareness, and as an emotion-focused strategy helps spending more time with family [30].

The British Medical Association, surveyed 2030 UK doctors from different specialties and medical students on their experience of racism in the medical profession [31]. Twenty-three per cent of 1239 who answered the questions mentioned they had considered leaving their job because of racial/ethnic discrimination, and 9% already left in the past two years. Black, Asian and other backgrounds were most likely to have considered leaving or had left their job. A total of 60% of the respondents said that the racial/ethnic harassment they experienced had negatively affected their wellbeing. However, there was no study in the last 5 years from the UK or Europe that specifically studied racial discrimination in surgery.

Overall, surgical residents who are discriminated, abused, or harassed at least a few times per month were most likely to have symptoms of burnout and suicidal thoughts [17]. Primary suicide risk factors are physical and emotional overload, the burden of malpractice lawsuits and regulatory bodies [32]. The burnout rate in orthopedic surgeons is 30–40%, and greater than 50% in residents. The rate of physician burnout was consistent with national estimates of approximately 50%, and it was found that 4.5% of general surgery residents had suicidal thoughts [17]. The mean suicide rate among surgeons was found to be 13.3% or doubles that of the general population [32].

The Association of Surgeons in Training in the UK created a new council role as equality and diversity officer, followed by other associations such as The British Orthopedic Trainee Association. One of the roles of the equality and diversity officers is to work on raising the awareness towards surgical training amongst underrepresented groups and to promote excellence in surgical training for the benefit of patients and trainees irrespective of race, gender, disability, sexual orientation, and religion. We believe the first step to work against such misconduct is to encourage victims to speak up and to seek guidance, when escalation takes place and formal report will be needed. We believe that each case should be studied, and offenders need to understand the potential consequences of such misconduct in forms of being suspended or losing license to practice. These suggestions for consequences of serious misconduct need support from different institutions and healthcare systems. In agreement, the UK General Medical Council published a statement supporting zero-tolerance to racism in the workplace.

To reduce racial/ethnic discrimination in surgery, wrongdoers can be confronted by means of restorative justice [27] which is based on an understanding that racial/ethnic conduct is a violation of people and relationships [20]. Furthermore, restorative justice is based on principles of respect, compassion and inclusivity. This means that offenders take responsibility for their actions and try to repair the harm they have done, for example by apologizing in public, or doing community service. The goal of restoring wrongdoing is to inspire meaningful commitment and accountability by providing an opportunity for healing, amendment, and rehabilitation. To handle bullying/racial discrimination in small groups between insulted and offender can look like this: Small groups are, facilitated by a trained advisor, who helps racial/ethnic discriminators to identify reasons behind their misconduct. All involved in the racial discrimination misconduct may be included and victims may describe how they were affected, and offenders may apologize.

Another remedy to deal with racism is to educate the surgical staff to fully understand the life experiences of surgical residents as well as to care about providers holistically, and to scrutinize the roots of distress of provider discrimination on surgical care delivery, along with supervising resident’s mental-health wellbeing to avoid burnout and/or attrition [18]. Individuals and medical organizations play an active role in the mitigation of racial bias experiences and associated burnout; both offenders and allies must be addressed [33]. At an individual level, value and respect must be highly recommended and microaggressions should be pointed out in a non-accusatory manner, as proposed by the GRIT method (gather, restate, inquire, talk it out) mnemonic [33]. Therefore, institutional leaders must increase their awareness of evidence of racial/ethnic discrimination in surgery in case they strive to cultivate a non-toxic diverse surgical environment.

We discussed race/ethnic discrimination’s negative health and psychological influence, as racial bias diminishes empathy, increases distrust, and reduced referral rates for specialty care for persons of color [21, 34]. It is also important to educate surgeons and staff members that this kind of negative impact has bearings on a whole surgical team performance as well as affects the costs of the health system. Racial health disparities are related to significant annual economic losses nationally, containing an estimated $35 billion in excess health care expenditures, $10 billion in illness-related lost productivity, and nearly $200 billion in premature deaths. Concerted efforts to reduce health disparities could, thus, have enormous economic and social value [35].

### Limitation and strength of the study

Self-report is most used to quantify experiences of racial discrimination. Potential limitations consist of self-ratings, although self-ratings are recommended for assessments of personality traits. As with any questionnaire, participants may deliver the answers they believe they should. On one hand, we do not know if subjective intuitions satisfactorily represent factual events. On the other hand, the private experience is likely to be more closely related to the own brain compared with that of an observer. Yet, one strength of Likert scales is claimed to be their capacity to show how strongly a participant feels about something. This, therefore, gives more details than a simple yes or no answer and, thus, do not provide in-depth replies and are easy to analyze statistically. A previous study [13] evaluated the MERSQI instrument and our results agree fairly well with their results. Their interrater reliability for overall scores ranged from 0.68 to 0.95 and our two correlations were r [10] = 0.78; *p* < 0.01, 2-tailed and r_s_ [10] = 0.71; *p* = 0.02 and significant. Most problems with interrater reliability were connected with restriction of alternative for study design and response rate while surveys were most often distributed electronically. Our median overall MERSQI score was 11 and mean 11.3 (range from 9 to 13.5 out) and Cook et al.’s [13] median overall MERSQI score was 11.3.

### Future directions

Most of the studies about racial discrimination in surgery during the last 5 years were performed in USA. Therefore, we lacked reports from the European healthcare systems or from the UK despite the continuous increase of racial/ethnic diversity in Europe. Future research should focus on disclosure of hotspots in surgery and its subspecialities where racial/ethnic discrimination is frequent. Consequently, a standardized pathway for reporting misconduct escalation should be developed with the support of trainees’ associations and healthcare institutions to guide victims of racial discrimination and encourage them to speak up to create mentally healthier surgeons with a brighter future in clinical practice.

## Conclusion

There was sufficient evidence for the presence of racial discrimination in surgical practice in the last 5 years. Ways to decrease racial discrimination in surgery exist. Healthcare and training systems must increase the awareness of these issues to eliminate the harmful effects on the individual level as well as on the surgical team performance. The discussion of the mentioned interpersonal complications needs to be extended to numerous countries with diverse healthcare systems.

## Data Availability

The corresponding author holds the data extracted from included studies, the data used for analyses. Materials used in the review are not publicly available.

## Declarations

### Review protocol

The review protocol is not publicly available.
